# Annotations

**Published:** 1896-06-13

**Authors:** 


### ANNOTATIONS.

### Modes of Heredity.

How people inherit diseases has been almost as much discussed as the cognate question how they inherit peculiarities. So long as it was maintained that acquired peculiarities, whether of body or of mind, could be transmitted, the transmission of a disease could be almost taken for granted. But the two hypotheses, so closely linked by the thought at root of them, fell together. The knowledge, however, that certain diseases are caused by micro-organisms raises another question, viz., to what extent and by what path may a microbic infection be transmitted from parent to offspring? This is fully discussed in a paper by Dr. Buckmaster in *Science Progress* for this month. The evidence that bacteria may be transferred from parent to offspring seems incontestable, and the routes by which this is possible appear to be two, viz., by a direct bacterial invasion of the essential reproductive cells, or by the egg cell or the embryo receiving micro-organisms from the female, in which case the blood-stream is the channel of infection. Bacterial infection then may be either germinative or placental, and in mammals the latter mode is not infrequently observed. The observations of Pasteur in regard to pébrine, a disease of silkworms, are well known. They showed that in this malady definite sporocyst forms were transmitted from the imago, or perfect insect, by way of the egg cell, and that the larva was directly infected in this manner. Gärtner also has absolutely demonstrated the fact that ova may contain pathogenic organisms. Infection from the male, however, stands on rather a different footing, and both on theoretical and experimental grounds it may be considered "that a germinative infection of the ovum never occurs by the conveyance of micro-organisms in the male reproductive cells."

### "Box Begging."

"Viator," in Monday's *Times*, asks "How much longer will the London public put up with the 'box-begging' nuisance?" . . . On four occasions within a space of two hundred yards he had a begging-box rattled in his face, and a "young lady" on each occasion solicited his alms for some charity or other. "It would be well," he continues, "if this mode of collecting funds for charities were abandoned." The present seems a favourable moment for stating that a good many of the recognised charitable institutions seriously deprecate this mode of raising funds for charities. Among the rest, the Hospital Sunday Fund discountenances it as much as possible. There are many reasons which, if the public would but think of them, make such a method of collecting money extremely undesirable. First and foremost is the fact that so many other agencies, including the Hospital Sunday Fund itself, do the work so much better, so much more economically, and with so much greater certainty of avoiding the possibility of mischance or peculation, that one is astonished to find street begging countenanced at all. The Sunday Fund collects its enormous sums at the cost of a fraction per cent. Its agents are the clergy and ministers of religion, the churchwardens and church managers of the metropolis, working for the love of the work, and the very pick and flower of philanthropy and high motive. Compared with this method of collection, and with these agents, what possibilities of wrong-doing lie open to view in the innumerable street collections, with their little-known agents, and the practical certainty that if there are not already, yet soon there will be, persons who will try to foist bogus institutions, in the name of charity, upon the public, and who may collect large sums in this dishonest way. We should vastly like to see much of the money now given to street collections put into the church-plates on Sundays; but, since many give who do not attend church, we advise that they take the trouble of sending their contributions either to the offices of the Sunday Fund direct, or to the treasurers of the recognised hospitals.

### Anti-vivisection Literature.

Periodical literature is responsible for much, and, among other things, for a far too frequent prostitution of knowledge and professional position to the exigencies of realism. The short story of the modern monthly must, above all things, seem real, and stuff which from its nature is offensive and from its style contemptible runs a chance of being accepted if only the false facts which it details can be made to appear real, and the false suggestions probable, by the tale being written by someone who ought to know. No doubt an editor of monthly fiction would lick his lips if he could persuade a barrister to represent English justice as corrupt and venal, and, by signing his name to the story, to give an air of truth to the insinuation; a tale of clerical scandal also would gain in piquancy and in apparent realism if signed by some well-known divine; and we may be pretty sure that a tale of human vivisection, a wild imagining according to which a wretched man is led by false representations to allow an experiment to be performed on him, and then, when he is once in the hands of the "Professor" and safely under chloroform, is calmly cut up alive, a story obviously and utterly false both in its facts and its suggestions, gains greatly in trade value, and equally in its full flavour of wickedness, by the fact that a qualified medical practitioner has been found sufficiently oblivious of all that is due to her profession to sign her name to it, and by the use of her name to give an air of truth to what she knew at every stroke she penned was false. That a periodical should publish such a thing is, perhaps, only a sign of the sensation-seeking times in which we live, but that a member of the medical profession, with a certain parade of physiological knowledge, and of the details of modern operating rooms, should put her name to such ghastly ravings of the imagination as we find in last month's *Ludgate Monthly* signed by Arabella Kenealy, is a matter which must be regretted and condemned by all who have at heart the honour of the profession. Falstaff says "What is honour? A word. What is in that word honour? What is that honour? Air." But the honour of the profession should be dear to its members.

